# Metabolic phenotype of B cells from young and elderly HIV individuals

**DOI:** 10.1186/s12979-021-00245-w

**Published:** 2021-08-21

**Authors:** Daniela Frasca, Suresh Pallikkuth, Savita Pahwa

**Affiliations:** 1 Department of Microbiology and Immunology, University of Miami Miller School of Medicine, RMSB 3146A 1600 NW 10th Ave, FL 33136 Miami, USA; 2 Sylvester Comprehensive Cancer Center, University of Miami Miller School of Medicine, Miami, FL USA

**Keywords:** Aging, HIV, B cells, Inflammation, Metabolism

## Abstract

**Background:**

HIV infection induces inflammaging and chronic immune activation (IA), which are negatively associated with protective humoral immunity. Similar to HIV, aging is also associated with increased inflammaging and IA. The metabolic requirements of B cell responses in HIV infected (HIV+) individuals are not known, although metabolic abnormalities have been reported in these individuals. How these metabolic abnormalities are exacerbated by aging is also not known.

**Methods:**

B cells were isolated by magnetic sorting from the blood of young and elderly HIV + individuals, as well as from the blood of age-matched healthy controls. We evaluated the composition of the B cell pool by flow cytometry, the expression of RNA for pro-inflammatory and metabolic markers by qPCR and their metabolic status using a Seahorse XFp extracellular flux analyzer.

**Results:**

In this study we have evaluated for the first time the metabolic phenotype of B cells from young and elderly HIV + individuals as compared to those obtained from age-matched healthy controls. Results show that the B cell pool of HIV + individuals is enriched in pro-inflammatory B cell subsets, expresses higher levels of RNA for pro-inflammatory markers and is hyper-metabolic, as compared to healthy controls, and more in elderly versus young HIV + individuals, suggesting that this higher metabolic phenotype of B cells is needed to support B cell IA. We have identified the subset of Double Negative (DN) B cells as the subset mainly responsible for this hyper-inflammatory and hyper-metabolic profile.

**Conclusions:**

Our results identify a relationship between intrinsic B cell inflammation and metabolism in HIV + individuals and suggest that metabolic pathways in B cells from HIV + individuals may be targeted to reduce inflammaging and IA and improve B cell function and antibody responses.

## Background

HIV infection induces low-grade systemic inflammation, inflammaging [1] and chronic immune activation (IA), which are negatively associated with immune responses through functional impairment of B cells, T cells, monocytes, NK and dendritic cells. With the use of combination anti-retroviral therapy (cART) IA decreases, but chronic IA still persists even in cART-treated virally suppressed individuals. The IA-driven dysregulation of the immune system is considered to be a major contributing factor in HIV disease pathogenesis, immune dysfunction and non-AIDS co-morbidities [2, 3].

Aging, similar to HIV, is also associated with increased inflammaging and IA [1]. Data from our laboratory have shown that ex vivo isolated B cells from elderly individuals express higher levels of TNF-α mRNA than those from younger controls [4, 5], and we have hypothesized and demonstrated that this “pre-activated” phenotype of the old B cells makes them refractory to further stimulation to generate protective antibody responses to the influenza vaccine in humans [5]. To confirm our hypothesis, we have also shown that blocking TNF-α by adding an anti-TNF-α antibody to B cells before stimulation significantly increases in vitro class switch in B cells from elderly individuals restoring it the levels observed in B cells from younger controls.

Immune cell function is highly dependent on the metabolic environment which is known to regulate the cell’s metabolic status. Immune cells need to perform a metabolic reprogramming to meet the bioenergetic and biosynthetic demands associated with inflammaging and IA, and rely on anaerobic glycolysis and oxidative phosphorylation (OXPHOS) to do so. During glycolysis, glucose is incompletely oxidized in the cytosol with lactate as the final product. During OXPHOS, carbon substrates such as glucose-derived pyruvate, fatty acids and glutamine are oxidized in the mitochondria to generate ATP. In HIV + individuals, metabolic abnormalities have been reported and associated with the effects of the drugs themselves as well as with the irreversible damage of metabolic tissues that started before the initiation of the treatment [6]. How these metabolic abnormalities are exacerbated by aging is not fully understood. It has been hypothesized that the age-associated changes in metabolism [7], increased metabolic stress [7, 8] and reduced clearance of metabolic waste [9] may represent an additional source of inflammatory stimuli driving inflammaging and IA.

In this study we have evaluated the metabolic profile of B cells isolated from the blood of young and elderly HIV infected (HIV+) individuals, as compared to those isolated from the blood of age-matched healthy controls. We show that B cells from both young and elderly HIV+, as compared to healthy controls, are enriched in pro-inflammatory B cell subsets, express higher levels of RNA for pro-inflammatory markers and are hyper-metabolic, and more in elderly HIV + versus young HIV+. We also show that the subset of Double Negative (DN) B cells, which represents the most pro-inflammatory B cell subset [4, 10], is the major contributor to this hyper-inflammatory and hyper-metabolic profile. We hypothesize that, similar to what we have observed in healthy elderly, this higher metabolic phenotype of B cells from HIV + individuals is needed to support IA. Our results suggest the intriguing possibility that metabolic pathways in B cells from HIV + individuals may be targeted to limit inflammaging and IA and improve B cell function and antibody responses in this vulnerable population.

## Results and discussion

### Composition of the peripheral B cell pool of young and elderly HIV + individuals, as compared to age-matched healthy controls

**Fig. 1:**
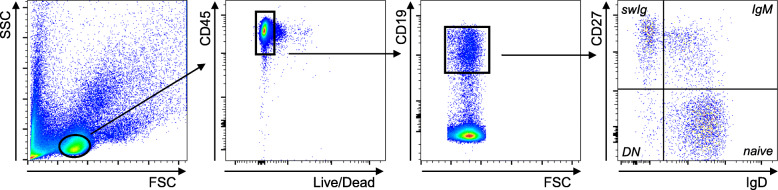
Gating strategies and a representative dot plot to show B cell subsets. PBMC (2 × 10^6^/ml) from one young healthy individual were stained for 20 min at room temperature with anti-CD45, anti-CD19, anti-CD27 and anti-IgD fluorochrome-conjugated antibodies to evaluate naive (IgD + CD27-), IgM memory (IgD + CD27+), switched memory (swIg, IgD-CD27+), and DN (IgD-CD27-) B cells

We have evaluated the composition of the peripheral B cell pool of young and elderly HIV + individuals, as compared to age-matched healthy controls, and measured the frequencies of the major B cell subsets. Briefly, we used anti-IgD and anti-CD27 antibodies to identify the four major subsets of B cells: naïve (IgD + CD27-), IgM memory (IgD + CD27+), switched memory (swIg, IgD-CD27+), and DN (IgD-CD27-) B cells. Results are shown in Table 1. Representative dot plots showing gate strategies and population names are shown in Fig. 1. In healthy individuals, aging induces a significant decrease in the frequencies of switched memory, a significant increase in the frequencies of naïve and DN and no change in IgM memory, confirming our previously published findings [4, 10], and similar effects of aging were also observed in HIV + individuals. Our results also clearly show that HIV infection induces a significant decrease in the frequencies of switched memory B cells and a significant increase in the frequencies of DN B cells in both young and elderly individuals. Interestingly, DN B cell frequencies in young HIV + individuals were higher than those in healthy individuals, both young and elderly, and this effect of HIV was further increased by aging. These latter results are slightly different from those we have previously published [11], likely due to the different staining protocol used to evaluate switched and IgM memory B cells, versus that used to evaluate activated and resting memory B cells.

**Table 1:** Composition of the B cell pool of recruited participants

B cell subsets	Healthy		HIV+	
	Young	Elderly	Young	Elderly
	(*n*=6)	(*n*=6)	(*n*=6)	(*n*=6)
Naive	52.7±2.4	62.7±3.6*	46.0±2.0	35.8±2.7* ^###^
IgM memory	21.2±7.7	16.2±3.3	24.3±1.2	15.7±3.8
Switched memory	21.8±0.9	4.0±1.6****	7.0±0.8^####^	7.5±1.9
Double Negative	4.5±1.1	14.7±2.3**	22.7±1.6^####^	40.9±3.3** ^###^

Asteriscs indicate differences between young and elderly participants, either healthy or HIV+: *p<0.05; **p<0.01; ****p<0.0001

Pound signs indicate differences between healthy and HIV+ participants, both young and elderly: ^###^*p*<0.001; ^####^*p*<0.0001

The increase in the frequencies of DN B cells has already been observed in individuals with inflammatory conditions such as healthy aging [4, 10, 12, 13] and obesity [14], as well as in patients with autoimmune diseases [15–20], chronic (HIV) [21] and acute (SARS-CoV-2) [22] viral infections, suggesting that these cells likely expand in vivo after chronic exposure to autoantigens or pathogen-derived antigens. However, the combined effects of aging and HIV on the frequencies of DN B cells, as well as on the frequencies of the other B cell subsets, have not been previously reported and we believe that our results represent an important contribution to the field of aging and HIV. The subset of DN B cells is the most pro-inflammatory B cell subset [14, 23], characterized by the expression of RNA for multiple markers of the senescence-associated secretory phenotype (SASP), most of which are markers of IA, without any stimulation. The levels of expression of SASP markers in ex vivo isolated B cells is known to be negatively associated with the capacity of B cells to secrete protective antibodies in response to infections and vaccination, as we have previously shown [5, 14, 24].

### B cells from young and elderly HIV + individuals, as compared to those from age-matched healthy controls, are characterized by higher expression of pro-inflammatory markers

**Fig. 2:**
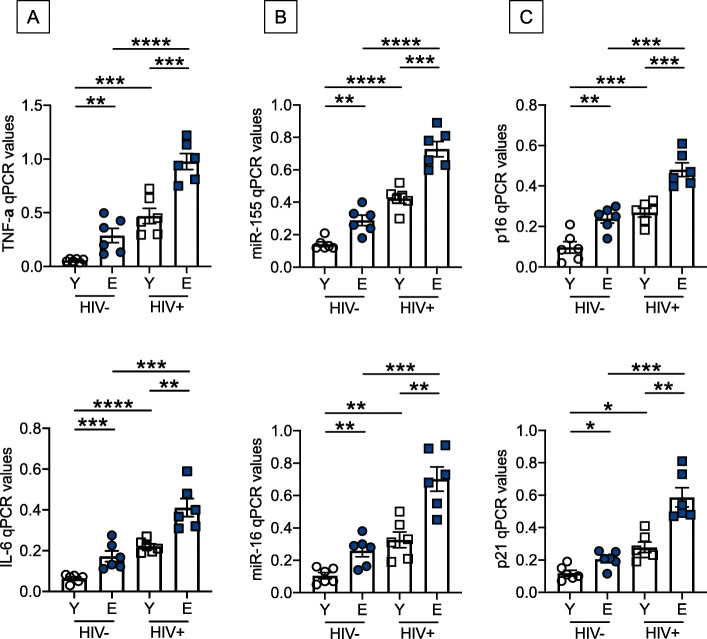
B cells from young and elderly HIV individuals, as compared to those from age-matched healthy controls, are characterized by higher expression of pro-inflammatory markers. B cells were isolated by magnetic sorting from the blood of young and elderly HIV individuals, and from age-matched healthy controls. B cells were left unstimulated, the RNA was extracted and qPCR performed to evaluate RNA expression of pro-inflammatory cytokines (**a**), pro-inflammarory miRs (**b**) and cell cycle inhibitors (**c**). *p < 0.05, **p < 0.01, ***p < 0.001, ****p < 0.0001

We next evaluated the expression of RNA for several SASP markers in unstimulated B cells isolated from the blood of young and elderly HIV + individuals, and from age-matched healthy controls. After isolation with magnetic beads, B cells were left unstimulated and then resuspended in TRIzol. The RNA was extracted and the expression of SASP markers evaluated by qPCR. We measured RNA expression of pro-inflammatory cytokines (TNF-α, IL-6) and micro-RNAs, miRs (miR-155, miR-16), as well as of markers of senescence and proliferation arrest (p16^INK4^ and p21^CIP1/WAF1^). These markers were selected because they were found negatively associated with class switch and secretion of influenza vaccine-specific antibodies in our previously published B cell studies [4, 5, 14, 25]. Figure 2 a-c show significantly higher expression of RNA for all these SASP markers in unstimulated B cells from elderly as compared to younger individuals, confirming and extending our previously published findings [5, 14, 24, 25]. SASP markers are also expressed at higher levels in B cells from both young and elderly HIV + individuals, as compared to healthy controls, and more in elderly than young HIV+, clearly indicating that HIV infection induces B cells with a senescent phenotype. Our results herein show for the first time the combined effects of aging and HIV on the expression of RNA for SASP markers in unstimulated B cells. Although B cells from healthy elderly and HIV + young and elderly individuals express high levels of RNA from markers associated with the SASP and with IA, they are transcriptionally and metabolically active, characterized by a high secretory phenotype leading to the secretion of several pro-inflammatory molecules, as well as autoimmune antibodies, as previously reported by us [23] and by others [26]. Importantly, we observed that RNA levels of all these SASP markers were comparable in B cells from healthy elderly individuals and in B cells from young HIV + individuals, suggesting that HIV infection accelerates age defects in B cells. Our results herein confirm our previously published observations, in which we used a single cell, targeted multiplex gene expression platform and predictive modeling to show that influenza-specific B cells exhibit divergent gene signatures in HIV+, ART-suppressed, influenza vaccinated individuals as compared to age-matched healthy controls [27]. These signatures identified 4 major IA pathways driven by the phosphatase and tensin homolog PTEN, with the PTEN-mediated inhibition of PI3K signaling pathway playing a crucial role in persistent IA and B cell dysfunction in HIV individuals.

**Fig. 3:**
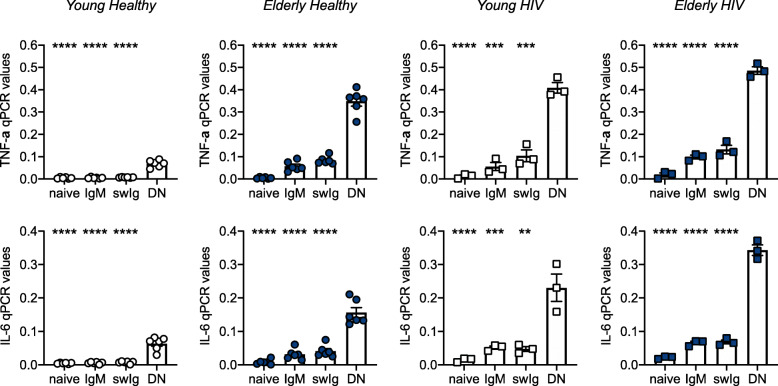
DN B cells express the highest levels of RNA for pro-inflammatory cytokines, as compared to the other B cell subsets. B cell subsets were sorted from the blood of young and elderly HIV individuals, and from age-matched healthy controls. After sorting, B cell subsets were left unstimulated, the RNA was extracted and qPCR performed to evaluate RNA expression of TNF-α (top) and IL-6 (bottom). Asteriscs indicate the differences between DN B cells and the other B cell subsets. **p < 0.01, ***p < 0.001, ****p < 0.0001

As shown in Table 1, DN B cell frequencies are significantly increased in the blood of healthy elderly and HIV + young and elderly individuals, as compared to healthy young individuals. We therefore evaluated if the SASP phenotype of unstimulated B cells from these individuals reflects increased DN B cell frequencies, the most pro-inflammatory B cell subset, and we measured the expression of the pro-inflammatory cytokines TNF-α and IL-6 in sorted B cell subsets. Results in Fig. 3 show that the RNA for TNF-α and IL-6 is expressed at significantly higher levels in DN B cells as compared to the other B cell subsets in the four groups of individuals, and more in healthy elderly and in HIV + young and elderly individuals, suggesting that DN B cells are the major contributors to the pro-inflammatory profile seen in unstimulated B cells.

### B cells from young and elderly HIV individuals, as compared to those from healthy controls, are hyper-metabolic and express higher levels of mRNA of enzymes involved in metabolic pathways

**Fig. 4:**
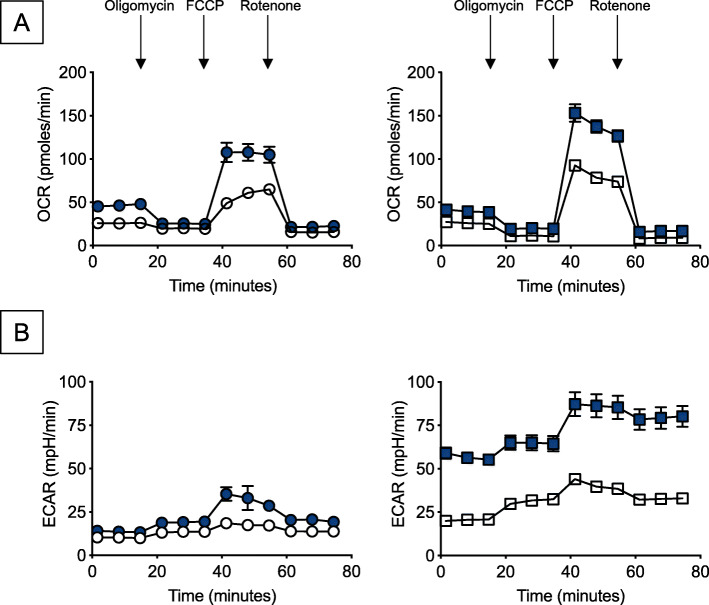
B cells from young and elderly HIV individuals, as compared to those from healthy controls, are hyper-metabolic as evaluated by higher OCR and ECAR. B cells, isolated as described in Fig. 2, were left unstimulated. B cells were seeded into the wells of an extracellular flux analyzer at the concentration of 2 × 10^5^/well in triplicate and run in a mitostress test. OCR results (**a**) and ECAR (**b**) results are shown. Open circles, young healthy donors; filled circles, elderly healthy donors; open squares, young HIV + individuals; filled circles, elderly HIV + individuals

Our preliminary results on gene expression have shown that ex vivo isolated B cells from healthy elderly individuals, characterized by higher expression of multiple SASP markers as compared to younger controls, are also hyper-metabolic and they up-regulate RNA expression of enzymes involved in OXPHOS and anaerobic glycolysis (Frasca et al., manuscript submitted), suggesting that B cells from elderly individuals rely on this hyper-metabolic phenotype to fulfil the demands for energy and biosynthetic precursors associated with higher IA. Therefore, we evaluated the metabolic profile of B cells from young and elderly HIV + individuals, as compared to B cells from age-matched healthy controls, using the mitostress test and Seahorse technology. This technology allows the real-time evaluation of changes in oxygen consumption rates (OCR) and extracellular acidification rates (ECAR), measures of OXPHOS and of anaerobic glycolysis, respectively. Figure 4 a and b show, as expected, higher OCR and ECAR in B cells from elderly as compared to younger controls. Moreover, we found higher OCR and ECAR in both young and elderly HIV + individuals, as compared to respective healthy control groups, with the highest values in the elderly HIV + compared to young HIV+. Similar to RNA expression of SASP markers, OCR and ECAR measures were comparable in B cells from healthy elderly individuals and in B cells from young HIV + individuals. These results demonstrate that B cells with high expression of IA markers have significantly higher OCR and ECAR as they rely on a more robust glycolytic pathways to support their IA phenotype. We can assume that the higher OCR and ECAR of B cells from elderly individuals, as well as from HIV + individuals, is associated with higher frequencies of DN B cells in the blood of these individuals. Our results in mice have indeed shown that the subset of Age-associated B Cells, ABCs, that increase in frequencies and numbers in the spleens of old mice and represent the mouse equivalent of human DN B cells, are hyper-inflammatory and hyper-metabolic [28]. We have preliminary evidence (unpublished) that even if these cells are in a “pseudo-activated” status, they further increase OCR and ECAR after mitogen stimulation. We believe that the evaluation of the metabolic profile of B cells prior to any stimulation is crucial to predict their capacity to respond to in vivo/in vitro stimulation.

**Fig. 5:**
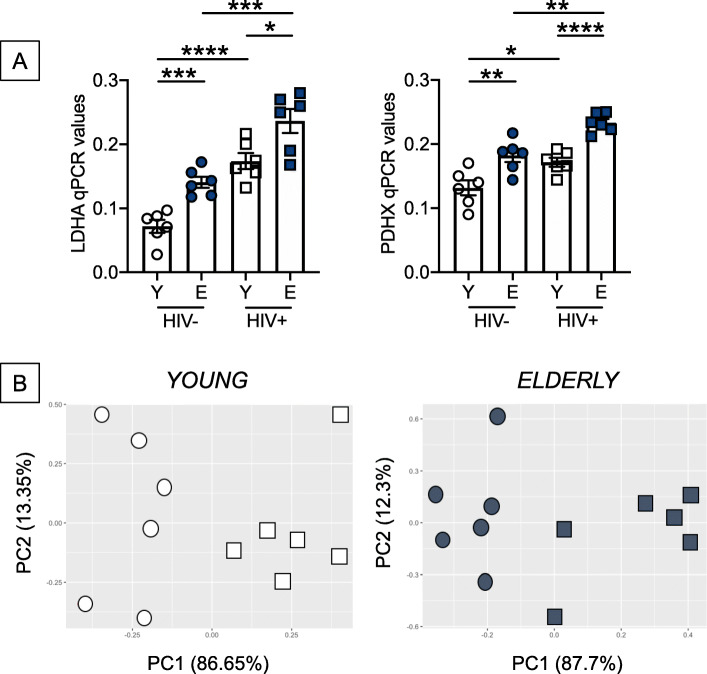
B cells from young and elderly HIV individuals, as compared to those from healthy controls, express higher levels of mRNA of enzymes involved in metabolic pathways. B cells, isolated as described in Fig. 2, were left unstimulated. **a** The RNA was extracted and qPCR performed to evaluate RNA expression of LDHA (left) and PDHX (right). *p < 0.05, **p < 0.01, ***p < 0.001, ****p < 0.0001. **b** PCA analyses with the axes showing the percentage of variation explained by PC1 and PC2. Each symbol corresponds to one individuals. Open circles, young healthy donors; filled circles, elderly healthy donors; open squares, young HIV + individuals; filled circles, elderly HIV + individuals

We confirmed the Seahorse results measuring mRNA levels of the enzymes involved in OXPHOS and anaerobic glycolysis, i.e. LDHA (Lactate Dehydrogenase) that converts pyruvate into lactate, and PDHX (Pyruvate Dehydrogenase), that converts pyruvate into acetyl-CoA, used in the Kreb cycle, respectively. Results in Fig. 5 a show increased mRNA expression of both enzymes in unstimulated B cells from healthy elderly versus young individuals, in B cells from both young and elderly HIV individuals as compared to healthy controls, and in B cells from elderly versus young HIV+. Levels of these metabolic enzymes were found comparable in B cells from healthy elderly individuals and from young HIV+, confirming once again that HIV accelerates age defects in B cells. Figure 5b shows PCA analyses and distinct clustering of LDHA and PDHX in B cells from healthy controls versus HIV + individuals, both young and elderly. These results are the first showing that the HIV-induced IA in B cells is associated with a hyper-metabolic profile.

**Fig. 6:**
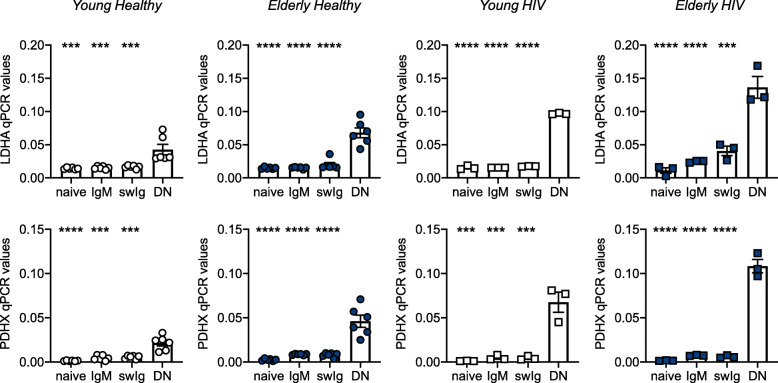
DN B cells express the highest levels of mRNA for metabolic enzymes, as compared to the other B cell subsets. B cell subsets were sorted and left unstimulated, the mRNA was extracted and qPCR performed to evaluate RNA expression of LDHA (top) and PDHX (bottom). Asteriscs indicate the differences between DN B cells and the other B cell subsets. ***p < 0.001, ****p < 0.0001

We also measured mRNA expression of LDHA and PDHX in unstimulated B cell subsets isolated from the blood of HIV + young and elderly individuals, as compared to healthy controls. Results in Fig. 6 show that both LDHA (top) and PDHX (bottom) mRNA levels are higher in DN B cells as compared to the other B cell subsets in the four groups of individuals. These results demonstrate that the subset of DN B cells is not only hyper-inflammatory but also hyper-metabolic. We hypothesize that this phenotype is needed to support B cell-intrinsic IA and the secretion of inflammatory products.

## Conclusions

HIV is associated with increased inflammaging and IA of B cells. Our results herein have identified a relationship between B cell IA and metabolism in HIV + individuals and have suggested that metabolic pathways in B cells from HIV + individuals may be targeted to reduce inflammaging and IA and improve B cell function and antibody responses. Further studies are needed to design effective strategies of intervention that may target the metabolic abnormalities related to increased inflammaging and IA of B cells. One approach would be to test an in vitro intervention using senolytic compounds that could help to improve B cell function and humoral immunity. This approach might represent a therapeutic strategy not only for HIV individusla but also for other inflammatory-based diseases.

## Methods

### Study participants

Experiments were performed using blood isolated from an established cohort of male and female young (30–50 years) and elderly (> 65 years) individuals, either healthy or with HIV, after appropriate signed informed consent and were approved with IRB protocols #20070481 and #20130399.

Healthy individuals participating in the study were screened for diseases known to alter the immune response or for consumption of medications that could alter the immune response (type-1 diabetes, congestive heart failure, cardiovascular disease, chronic renal failure, malignancies).

HIV + participants in this study were part of the aging & HIV study FLORAH (FLu Responses Of people in relation to Age and HIV). All the HIV + participants were on cART with virologic suppression (plasma HIV RNA < 40 copies/ml) for ≥ 1 year prior to study entry. Recruitmen of HIV- and HIV + individuals reflects the distribution of ethnicities present in the Miami Dade County, with 55 % non-Hispanic and 45 % Hispanic individuals. In each group, 50 % males and 50 % females have been recruited.

### PBMC collection

PBMC were collected using Vacutainer CPT tubes (BD 362761) and cryopreserved. PBMC (1 × 10^6^/ml) were thawed and maintained overnight in complete medium (RPMI 1640, supplemented with 10 % FCS, 10 µg/ml Pen-Strep, 1mM Sodium Pyruvate, and 2 × 10^–5^ M 2-ME and 2 mM L-glutamine). Then cells were washed, resuspended in FACS buffer and stained with the antibodies below.

### Flow cytometry

After thawing, PBMC (2 × 10^6^/ml) were stained for 20 min at room temperature with the following antibodies: anti-CD45 (Biolegend 368540), anti-CD19 (BD 555415), anti-CD27 (BD 555441) and anti-IgD (BD 555778) to measure naive (IgD + CD27-), IgM memory (IgD + CD27+), switched memory (IgD-CD27+), and DN (IgD-CD27-) B cells. Up to 10^4^ events in the B cell gate were acquired on an LSR-Fortessa (BD) and analyzed using FlowJo 10.0.6 software. Single color controls were included in every experiment for compensation. Isotype controls were also used in every experiment to set up the gates.

### B cell enrichment

After thawing, B cells were isolated from PBMC using magnetic CD19 Microbeads (Miltenyi), following manufacturer’s instructions. At the end of the purification procedure, cells were 90–95 % CD19-positive by cytofluorimetric analysis. Within 15 min of magnetic sorting, B cells were divided into 3 aliquots: one immediately resuspended in TRIzol (ThermoFisher Scientific) for RNA extraction or in lysis buffer for mRNA extraction with the µMACS mRNA isolation kit (Miltenyi), and one used for metabolic measurements within 1 h.

### B cell sorting

B cell subsets were sorted is a Sony SH800 cell sorter using anti-CD45, anti-CD19, anti-CD27 and anti-IgD antibodies. Cell preparations were typically > 98 % pure.

### RNA extraction and cDNA preparation

To evaluate RNA expression of pro-inflammatory markers (cytokines, miRs and cell cycle regulators), and of the enzymes involved in metabolic pathways, total RNA was extracted from unstimulated B cells, or from unstimulated B cell subsets, resuspended in TRIzol, according to the manufacturer’s protocol, resuspended in 10 µl of preheated H_2_O, and stored at -80 °C until use.

To evaluate RNA expression of the enzymes involved in metabolic pathways (LDHA, Lactate Dehydrogenase and PDHX, Pyruvate Dehydrogenase), the mRNA was extracted from unstimulated total B cells, or from unstimulated B cell subsets, using the µMACS mRNA isolation kit, according to the manufacturer’s protocol, eluted into 75 µl of preheated elution buffer, and stored at -80 °C until use.

Reverse Transcriptase (RT) reactions were performed in a Mastercycler Eppendorf Thermocycler to obtain cDNA. Briefly, 2 µl of RNA at the concentration of 0.5 µg/µl or 10 µl of mRNA were used as template for cDNA synthesis in the RT reaction. Conditions were: 40 min at 42 °C and 5 min at 65 °C. For miRs quantification, RT reactions were performed in the presence of specific primers.

### Quantitative PCR (qPCR)

Reactions were conducted in MicroAmp 96-well plates, and run in the ABI 7300 machine. We determined the cycle number at which transcripts reached a significant threshold (Ct) for each target gene, and for GAPDH or U6 as controls. The difference in Cts between the housekeeping genes (GAPDH or U6) and the target genes was calculated as ΔCt. Then the relative amount of the target gene was expressed as 2^−ΔCt^ and indicated as qPCR values. Reagents and primers for qPCR amplification were from ThermoFisher. Primers were: GAPDH, Hs99999905_m1; TNF, Hs01113624_g1; IL-6, Hs00985639_m1; IL-8, Hs00174103_m1; p16^INK4^ (CDKN2A), Hs00923894_m1; p21^CIP1/WAF1^, Hs00355782_m1; p53, Hs01034249_m1; LDHA, Hs01378790_g1; PDHX, Hs00185790_m1; U6, 001973; miR-155, 002623; miR-16, 000391.

### Metabolic measurements

OCR and ECAR were measured in a Mitostress test conducted in a Seahorse XFp extracellular flux analyzer (Agilent). Briefly, unstimulated ex vivo isolated B cells were seeded in a CellTAK (BD Biosciences)-coated plate, at the concentration of 2.5 × 10^5^/well. Cells were initially incubated in XF DMEM medium supplemented with glutamine, glucose and pyruvate (200 µL of each reagent in 20 mL of medium). Maximal respiratory capacity was measured by treating with Oligomycin (1 µM) to block ATP production, followed by the uncoupling agent FCCP (fluoro-carbonyl cyanide phenylhydrazone, 5 µM), to dissipate proton gradients and allow electron transport and oxygen consumption to operate at maximal rate. This elevated OCR is suppressed by Rotenone/Antimycin (1 µM), showing that respiration is mitochondrial. To confirm Seahorse results, we also analyzed the metabolic status of the B cells by qPCR gene expression analysis of LDHA and PDHX.

### Statistical analyses

To examine differences between 4 groups, two-way ANOVA was used. Group-wise differences were analyzed afterwards with Bonferroni’s multiple comparisons test, with p < 0.05 set as criterion for significance. To examine differences between 2 groups, Student’s t tests (two-tailed) were used. GraphPad Prism version 8 software was used to construct all graphs. Principal Component Analyses (PCA) were generated using RStudio Version 1.1.463.

## Data Availability

Data are available upon request to the corresponding authors.

## Abbreviations

ECAR: Extracellular acidification rate.
miRs: micro-RNAs.
OCR: Oxygen consumption rate.
SASP: Senescence-associated secretory phenotype.
